# Opaganib Downregulates N-Myc Expression and Suppresses In Vitro and In Vivo Growth of Neuroblastoma Cells

**DOI:** 10.3390/cancers16091779

**Published:** 2024-05-05

**Authors:** Lynn W. Maines, Staci N. Keller, Ryan A. Smith, Randy S. Schrecengost, Charles D. Smith

**Affiliations:** Apogee Biotechnology Corporation, 1214 Research Blvd, Suite 2015, Hummelstown, PA 17036, USA

**Keywords:** neuroblastoma, opaganib, ABC294640, sphingolipid, N-myc, sphingosine kinase

## Abstract

**Simple Summary:**

Neuroblastoma is the most common cancer in infants and the most common solid tumor outside the brain in children, and responds poorly to current therapies. The sphingolipid-modifying drug opaganib, which has anticancer and anti-inflammatory activity in many preclinical models, was tested for inhibition of neuroblastoma cell proliferation in cell culture and in tumors growing in mice. Opaganib inhibited cell proliferation regardless of the MYCN gene status of the neuroblastoma cells. Treatment of tumor-bearing mice with opaganib in combination with the established drugs irinotecan and temozolomide reduced tumor growth and increased survival compared with irinotecan plus temozolomide alone. Because opaganib has already demonstrated safety in patients with cancer, this new drug may provide improved therapy for neuroblastoma patients.

**Abstract:**

Neuroblastoma (NB), the most common cancer in infants and the most common solid tumor outside the brain in children, grows aggressively and responds poorly to current therapies. We have identified a new drug (opaganib, also known as ABC294640) that modulates sphingolipid metabolism by inhibiting the synthesis of sphingosine 1-phosphate (S1P) by sphingosine kinase-2 and elevating dihydroceramides by inhibition of dihydroceramide desaturase. The present studies sought to determine the potential therapeutic activity of opaganib in cell culture and xenograft models of NB. Cytotoxicity assays demonstrated that NB cells, including cells with amplified *MYCN*, are effectively killed by opaganib concentrations well below those that accumulate in tumors in vivo. Opaganib was shown to cause dose-dependent decreases in S1P and hexosylceramide levels in Neuro-2a cells, while concurrently elevating levels of dihydroceramides. As with other tumor cells, opaganib reduced c-Myc and Mcl-1 protein levels in Neuro-2a cells, and also reduced the expression of the N-Myc protein. The in vivo growth of xenografts of human SK-N-(BE)2 cells with amplified *MYCN* was suppressed by oral administration of opaganib at doses that are well tolerated in mice. Combining opaganib with temozolomide plus irinotecan, considered the backbone for therapy of relapsed or refractory NB, resulted in increased antitumor activity in vivo compared with temozolomide plus irinotecan or opaganib alone. Mice did not lose additional weight when opaganib was combined with temozolomide plus irinotecan, indicating that the combination is well tolerated. Opaganib has additive antitumor activity toward Neuro-2a tumors when combined with the checkpoint inhibitor anti-CTLA-4 antibody; however, the combination of opaganib with anti-PD-1 or anti-PD-L1 antibodies did not provide increased antitumor activity over that seen with opaganib alone. Overall, the data demonstrate that opaganib modulates sphingolipid metabolism and intracellular signaling in NB cells and inhibits NB tumor growth alone and in combination with other anticancer drugs. Amplified *MYCN* does not confer resistance to opaganib, and, in fact, the drug attenuates the expression of both c-Myc and N-Myc. The safety of opaganib has been established in clinical trials with adults with advanced cancer or severe COVID-19, and so opaganib has excellent potential for treating patients with NB, particularly in combination with temozolomide and irinotecan or anti-CTLA-4 antibody.

## 1. Introduction

Neuroblastoma (NB) is the most common cancer in children less than 1 year of age and the most common extracranial solid tumor in children, accounting for ~10% of all childhood cancers [1]. Approximately half of NB patients are diagnosed with low- or medium-risk, and these patients have excellent 5-year survival rates. Unfortunately, for the remaining patients diagnosed with high-risk NB, this survival rate is only ~50% [2,3]. High-risk NB usually occurs in children older than 18 months, frequently metastasizes to the bone, and is often associated with *MYCN* gene amplification (40–50% of cases) [4,5]. Amplification of *MYCN* strongly predicts a poorer prognosis for both tumor progression and overall survival [6,7,8], and, consequently, the protein product N-Myc is a prime target for new drugs for NB treatment [9,10,11]. Additionally, genes associated with activation of RAS-MAPK signaling are mutated in 65% of relapsed tumors [12], and overexpression of the anti-apoptotic proteins Bcl-2 and Mcl-1 is implicated in the poor response of NB to therapy [13,14]. Chemotherapy for high-risk NB patients begins with induction therapy using a battery of cytotoxic drugs, typically including platinum drugs, alkylators, and topoisomerase inhibitors [2,15,16]. Unfortunately, these agents are generally poorly effective and inflict considerable short- and long-term toxicity to the patient, including increased secondary cancers [17]. Therefore, a great deal of current effort is focused on identifying appropriate new targets for NB therapy through consideration of critical and/or aberrant pathways in this disease [18,19,20,21,22,23]. Difloromethylornithine (DFMO, elfornithine) has been studied as a possible modulator of N-Myc via alteration of polyamine levels [24], and has recently been approved by the FDA for maintenance therapy of high-risk NB [25,26]. Attempts to treat high-risk NB with antibodies against the checkpoint proteins PD-1, PD-L1, and CTLA-4 have not established therapeutic benefit [27]. Clearly, new and more effective therapies are desperately needed for NB patients, and significant effort is being focused on identifying new targets for NB drugs.

Sphingolipid metabolism is a key pathway in cancer biology in which ceramides, dihydroceramides (dhCer), sphingosine, and sphingosine 1-phosphate (S1P) regulate tumor cell death, proliferation, and drug resistance, as well as host angiogenesis, inflammation, and immunity (reviewed in [28,29,30,31]). In particular, sphingosine kinases (SK1 and SK2) are key regulators of the ceramide/S1P rheostat that controls tumor cell proliferation and death, as well as tumor sensitivity to radiation and chemotherapy (reviewed in [32,33,34]). Sphingosine kinases are frequently overexpressed in many cancers, and are essential for tumor cell survival and proliferation [29]. In parallel, dhCer desaturase (DES1) controls the ratio of saturated and unsaturated ceramides, and this regulates apoptotic and autophagic signaling in cancer cells [35,36]. Because of these roles of sphingolipids in cancer biology, chemical modulators of sphingolipid metabolism are potential candidates for new anticancer drugs.

Opaganib is an orally active, isozyme-selective inhibitor of SK2, and is competitive with respect to sphingosine [37,38]. Opaganib depletes S1P and elevates ceramide in tumor cells, suppresses signaling through pERK, pAKT, and NFκB, and promotes autophagy and/or apoptosis [29,37,38]. Opaganib also downregulates c-Myc in a variety of cell lines and reduces androgen receptor expression in prostate cancer cells. Because it acts as a sphingosine mimetic, opaganib also inhibits DES1, thereby increasing levels of dhCer and promoting autophagy in cells. Opaganib has antitumor activity in a broad range of mouse models [29,37,39] and anti-inflammatory activity in several rodent models [40,41,42,43,44]. In addition to single-agent cytotoxicity, opaganib has been combined with a variety of anticancer drugs in vitro and in vivo. In the present studies, the effects of opaganib on intracellular signaling and proliferation of NB cells were analyzed. Additionally, the ability of opaganib to suppress tumor growth in vivo was assessed as a single-agent and in combination with cytotoxic drugs used to treat NB or checkpoint antibodies.

## 2. Materials and Methods

### 2.1. Materials

Tumor cell lines (Neuro-2a, SK-N-SH, SK-N-AS, SK-N-MC, IMR32, SK-H-(BE)2, and Lewis lung carcinoma (LLC)) were purchased from the American Type Culture Collection and maintained in RPMI 1640 medium supplemented with 10% fetal bovine serum from Invitrogen (Carlsbad, CA, USA) and 100 units/mL penicillin-streptomycin. Geltrex was purchased from ThermoFisher Scientific (Waltham, MA, USA). Opaganib (GMP-grade) was synthesized according to French et al. [37] and dissolved in a vehicle consisting of 46.7% polyethylene glycol 400, 46.7% saline, and 6.6% EtOH. Temozolomide and irinotecan were purchased from Sigma-Aldrich (St. Louis, MO, USA). Antibodies for immunoblotting were purchased from Cell Signaling Technology (Danvers, MA, USA): Mcl-1 (catalog number 5453), c-Myc (catalog number 5605), N-Myc (catalog number 84406), GAPDH (catalog number 5174), Erk (catalog number 4695), and pErk (catalog number 4370). Anti-mouse PD-1 antibody, anti-mouse PD-L1 antibody, and anti-mouse CTLA-4 antibody were purchased from BioXCell (West Lebanon, NH, USA) and suspended in sterile phosphate-buffered saline (PBS) for intraperitoneal administration. SCID mice were purchased from the National Cancer Institute (Bethesda, MD, USA), while A/J and C57BL/6 mice were purchased from Jackson Labs (Bar Harbor, ME, USA). 

### 2.2. In Vitro Cytotoxicity and Signaling Assays

For cytotoxicity assays, cells were seeded in 96-well plates and 24 h later treated with 0 to 50 μM opaganib for 72 h. Cell viability was determined by a standard sulforhodamine B assay, as described previously [29]. In lipidomic analyses, ceramide species, sphingoid bases, and their phosphates were quantified by the Lipidomics Shared Resource at the Medical University of South Carolina following their validation using high-performance liquid chromatography–tandem mass spectrometry procedures. Results are expressed as the level of the sphingolipid normalized to protein levels measured using the Bradford assay. For protein expression studies, cell lysates were prepared using a buffer containing 25 mM Tris-HCl, 150 mM NaCl, 1% NP-40, 1% sodium deoxycholate, and 0.1% SDS. After centrifugation, cell lysates were normalized for protein content using the BCA assay (Pierce), resolved by SDS-PAGE, and transferred to PVDF membranes. Membranes were blocked with 10% bovine serum albumin and probed with the indicated primary antibodies at dilutions specified by the manufacturers. All immunoblots were visualized by enhanced chemiluminescence and quantified using ImageJ software (version IJ 1.54g) with normalization to GAPDH. 

### 2.3. In Vivo Tumor Growth Assays

Animal studies have been carried out in accordance with the Guide for the Care and Use of Laboratory Animals by the U.S. National Institutes of Health. In the first experiments, NOD/SCID mice (6–8 weeks old) were injected with 3 × 10^6^ SK-N-(BE)2 NB cells per mouse into the right hind flank subcutaneously on Day 0 of the experiment, and tumors were allowed to grow to ~100 mm^3^. Mice were then randomized into treatment groups (N = 8/group) and treated with either vehicle or 50 mg/kg opaganib given by oral gavage 5 days/week. For each mouse, body weight and tumor size were measured thrice per week, and tumor volumes were calculated by using the following formula: volume = 1/2 × length × width^2^. The toxicity of the treatments was assessed by careful observation of the mice for signs of distress, including respiratory difficulties, gastrointestinal distress, evidence of spastic paralysis, convulsions, or blindness. No mice displayed any of these abnormalities, so individual mice were euthanized by CO_2_ asphyxiation and cervical dislocation when the tumor volume reached ≥3000 mm^3^. 

Because the immune status of the host can affect tumor growth and response to therapy, the second tumor model utilized immunocompetent mice injected with syngeneic LLC cells. Specifically, C57BL/6 mice were injected with 10^5^ LLC cells suspended in PBS into the right hind flank subcutaneously on Day 0 of the experiment. When tumors reached ~150 mm^3^, mice were randomized into the following treatment groups (N = 7–8/group): control (vehicle only); opaganib alone (oral gavage at 50 mg/kg 5 days/week until sacrifice); irinotecan (IRIN, 5 mg/kg) plus temozolomide (TMZ, 25 mg/kg) given by intraperitoneal injection 5 days/week; or a combination of opaganib + IRIN + TMZ (50, 5, and 25 mg/kg, respectively). Mouse body weight, tumor growth, and potential toxicity were monitored, and mice were euthanized as indicated above.

In the third independent tumor model, the effects of opaganib alone and in combination with chemotherapy or immunotherapy were assessed using xenografts of Neuro-2a cells growing in immunocompetent syngeneic A/J mice. Specifically, male A/J mice were injected with 10^6^ Neuro-2a cells suspended in 100 µL PBS/Geltrex on the right hind flank. When tumors reached 150–450 mm^3^, mice were randomized into the following treatment groups (N = 9–10 mice/group): control (vehicle only); opaganib alone (oral gavage at 50 mg/kg 5 days/week until sacrifice); irinotecan (IRIN, 5 mg/kg) plus temozolomide (TMZ, 25 mg/kg) given by intraperitoneal injection 5 days/week; or a combination of opaganib + IRIN + TMZ (50, 5, and 25 mg/kg, respectively). In separate experiments, A/J mice bearing Neuro-2a tumors as above were randomized into the following treatment groups: control (vehicle only); opaganib alone (oral gavage at 50 mg/kg 5 days/week); anti-mouse PD-1, PD-L1, or CLTA-4 antibodies (200 μg injected intraperitoneally twice weekly for 3 weeks); or a combination of opaganib plus the antibody. Mouse body weight, tumor growth, and potential toxicity were monitored, and mice were euthanized as indicated above.

### 2.4. Statistics

Mouse survival rates were compared using the Kaplan–Meier approach with the Gehan–Breslow–Wilcoxon test using GraphPad Prism software (Version 5.0). Other data were analyzed by one-way ANOVA using the Tukey post hoc test. Differences are considered statistically significant at *p* < 0.05. Error bars in the figures represent the mean ± standard deviation of the treatment groups calculated with GraphPad Prism 5.

## 3. Results

### 3.1. In Vitro Effects of Opaganib on NB Cells

Cytotoxicity data for opaganib toward a panel of NB cell lines, including human *MYCN* single-copy cells (SK-N-SH, SK-N-AS, and SK-N-MC); human *MYCN* amplified cells (IMR32 and SK-H-(BE)2); and mouse *MYCN* single-copy cells (Neuro-2a), are indicated in Table 1. The data demonstrate that NB cells are killed by opaganib concentrations well below those that accumulate in tumors in vivo (~80 μg/g tissue, which is ~200 μM) [37]. Importantly, amplification of *MYCN* does not result in resistance to opaganib.

We have previously demonstrated in several cell types that opaganib reduces S1P levels and substantially elevates dihydroceramide levels, reflecting dual inhibition of SK2 and DES1 [37]. Additional lipidomic analyses were conducted on Neuro-2a cells treated with varying concentrations of opaganib for 24 h. As indicated in Table 2, acute treatment of Neuro-2a cells with opaganib reduced S1P and elevated total ceramide and dihydroceramide levels at 3 µM opaganib, which is near the cytotoxicity IC_50_ for these cells. Interestingly, higher opaganib concentrations also markedly decreased deoxyceramides and hexosylceramides in these cells, suggesting additional sphingolipid targets for opaganib.

**Figure 1:**
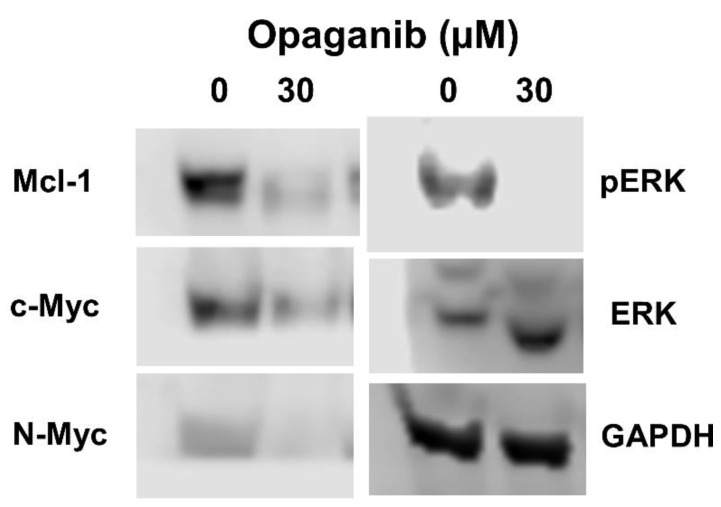
Effects of genetic and pharmacologic inhibition of SK2 on signaling proteins. Neuro-2a cells were incubated with 0 or 30 μM opaganib for 24 h, and the indicated proteins were examined by immunoblotting and quantified by ImageJ (version IJ 1.54g) analyses with normalization to GAPDH. Uncropped WB figures can be reviewed in Figure S1.

The effects of opaganib on intracellular signaling proteins in Neuro-2a cells were examined by immunoblotting (Figure 1). As with other tumor cells, treatment of Neuro-2a cells with opaganib decreased the expression of both c-Myc and Mcl-1 (48% and 70%, respectively) and completely eliminated pERK. Importantly, opaganib also reduced N-Myc protein expression in Neuro-2a cells (50%). Overall, treatment of NB cells with opaganib decreases proliferative signaling (c-Myc, N-Myc, and pERK) in concert with removing anti-apoptotic signaling (Mcl-1). 

### 3.2. In Vivo Effects of Opaganib on NB Tumors

Several in vivo tumor studies were conducted to evaluate the potential for treating NB patients with opaganib, including experiments using opaganib alone, in combination with irinotecan (IRIN) plus temozolomide (TMZ), or in combination with checkpoint antibodies.

#### 3.2.1. Single-Agent Opaganib

**Figure 2:**
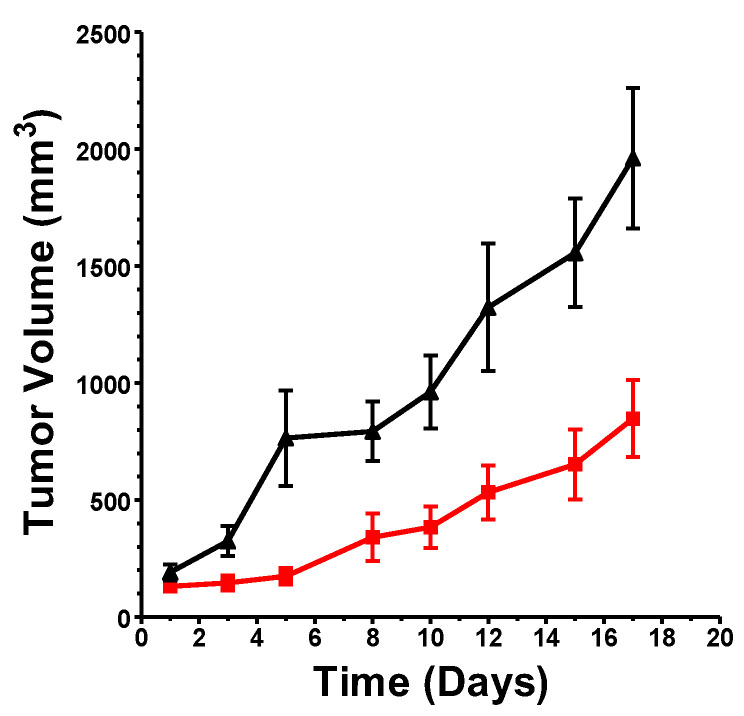
Antitumor activity of opaganib. SK-N-(BE)2 cells were implanted into NOD/SCID mice and allowed to grow to ~100 mm^3^. Animals were then treated orally with 0 (▲) or 50 (■) mg/kg opaganib 5 days/week, and body weight and tumor size were monitored. Values indicated the mean ± SEM for each treatment group (N = 8/group).

An initial study using xenografts of SK-N-(BE)2 NB cells in immunodeficient NOD/SCID mice demonstrated effective suppression of tumor growth by treatment with opaganib at 50 mg/kg/day, 5 days/week (Figure 2). Tumors in mice treated with vehicle progressed rapidly, necessitating euthanizing the animals in approximately 3 weeks. In contrast, mice treated with oral opaganib had substantially reduced rates of tumor growth, indicating antitumor activity against human NB cells with amplified *MYCN*. Further experiments were conducted in immunocompetent syngeneic mouse model systems because of their importance to the host immune response and the impact of opaganib treatment on immune responsiveness [45]. 

#### 3.2.2. Combination with Temozolomide and Irinotecan

We evaluated the antitumor activity of opaganib in combination with irinotecan (IRIN) plus temozolomide (TMZ) because IRIN + TMZ is the backbone for the treatment of recurrent or refractory NB. Opaganib (50 mg/kg) was administered by oral gavage 5 days/week, and IRIN and TMZ (5 and 25 mg/kg, respectively) were given by intraperitoneal injection, also in a 5 day on/2 day off schedule. Body weights and animal behavior and appearance were carefully monitored to detect possible overt toxicity from the drug combinations. Tumor volumes and body weights were measured daily for at least two full cycles of drug treatments. When tumor volumes reached >3000 mm^3^, animals were euthanized per IACUC requirements.

**Figure 3:**
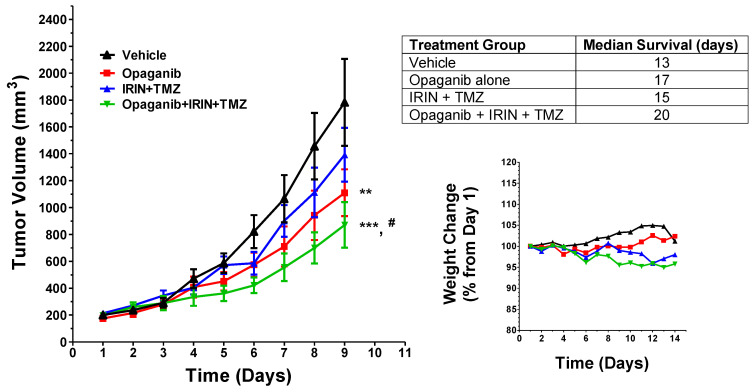
Effects of opaganib in combination with irinotecan and temozolomide on LLC tumor growth. Immunocompetent C57BL/6 mice bearing LLC tumors (*n* = 7 or 8/group) were treated with the following: vehicle; opaganib; IRIN + TMZ; or a combination of opaganib + IRIN + TMZ. (**Left** Panel): The average tumor volume of each group is shown for each day, and statistical comparisons on Day 9 are indicated (** *p* < 0.01 from the vehicle group, *** *p* < 0.001 from the vehicle group, ^#^ *p* < 0.05 from the IRIN + TMZ group). (**Right** Panel): The average body weights of mice in each treatment group are shown up to Day 14, completion of 2 full cycles of drug treatments. Table: Individual mice were sacrificed when the tumor volume reached >3000 mm^3^. The median survival in days is shown for each treatment group.

In the first study, male C57BL/6 mice were injected on the right hind flank with 10^6^ Lewis lung carcinoma (LLC) cells suspended in 100 µL of PBS/Geltrex. When tumors reached 150–200 mm^3^, mice were randomized into treatment groups of 7–8 mice/group and received the following: vehicle; opaganib alone; IRIN + TMZ; or opaganib + IRIN + TMZ. As shown in Figure 3, the LLC tumors in vehicle-treated mice grew very rapidly, and opaganib alone reduced tumor growth compared to the vehicle group (*p* < 0.01 at Day 9). Treatment with IRIN + TMZ induced only a minor, non-significant reduction in tumor growth. Importantly, the three-drug combination of opaganib + IRIN +TMZ showed a statistically significant reduction in tumor growth by Day 9 compared to either the vehicle group (*p* < 0.001) or the IRIN + TMZ group (*p* < 0.05), indicating that the three drugs in combination are more efficacious than the standard IRIN + TMZ treatment. Body weights were measured daily to evaluate the overall health of the mice and the potential toxicity of the multiple drug combination. As also shown in Figure 3, all treatment groups maintained similar body weights throughout two full cycles of 7-day drug treatments. Additionally, mice in all the treatment groups remained well-groomed and active throughout the study, indicating no excessive toxicity from combining the three drugs. Finally, survival time until tumors reached >3000 mm^3^ was tracked for all individual mice in the study. As shown in Figure 3 table, survival times inversely corresponded with the rate of tumor growth, with vehicle-treated mice having a median survival of 13 days. Opaganib treatment alone modestly improved survival, while opaganib + IRIN + TMZ treatment resulted in substantially greater median survival than vehicle- or IRIN + TMZ-treated mice (*p* < 0.01 for each comparison). Overall, this study demonstrated a significant increase in efficacy of opaganib + IRIN + TMZ relative to vehicle- and IRIN + TMZ-treated mice. Also of importance, the study indicates that there is no overt toxicity for the combination of opaganib + IRIN + TMZ over 2 weeks of treatment. 

**Figure 4:**
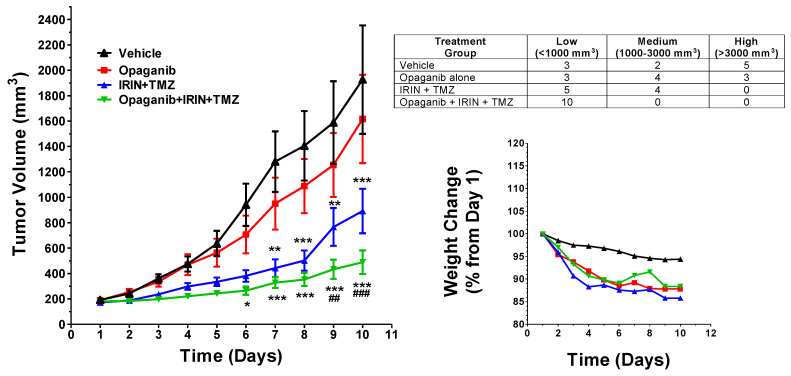
Effects of opaganib in combination with irinotecan and temozolomide on Neuro-2a tumor growth. Immunocompetent A/J mice bearing Neuro-2a tumors (*n* = 9 or 10/group) were treated with the following: vehicle; opaganib; IRIN + TMZ; or opaganib + IRIN + TMZ. (**Left** Panel): The average tumor volume of each group is shown for each day, and statistical comparisons on Day 9 are indicated (*, ** and *** *p* < 0.05, 0.01 and 0.001, respectively, from the vehicle group; ## and ### *p* < 0.01 and 0.001, respectively, from the IRIN +TMZ group). (**Right** Panel): The average body weights of mice in the study up to Day 10 are shown. Table: Tumor size on Day 10 was used to classify individual tumors as having low, medium, or high growth rates.

In the second study, Neuro-2a cells were used because they are a common model for NB tumors grown in immunocompetent syngeneic mice [46]. In these studies, male A/J mice were injected on the right hind flank with 10^6^ Neuro-2a cells suspended in 100 µL of PBS/Geltrex, and tumors were monitored until they reached 150–250 mm^3^. Mice were then randomized into treatment groups (N = 9–10 mice/group) and received: vehicle; opaganib alone; IRIN + TMZ; and opaganib + IRIN + TMZ at the same doses and schedule used in the LLC model. Tumor volumes and animal health were monitored as indicated above, and individual mice were euthanized when their tumor volume exceeded 3000 mm^3^ per IACUC requirements. As shown in Figure 4, the body weights of the vehicle-treated, tumor-bearing mice decreased approximately 5% in the first 10 days of the study, suggesting that this strain is more susceptible to cachexia than are C57BL/6 mice. Body weight loss increased slightly in all the drug-treated groups; however, this was not sufficient to necessitate the euthanasia of any of the animals. 

As indicated in Figure 4, the Neuro-2a tumors in vehicle-treated mice grew rapidly, necessitating the euthanasia of some mice beginning on Day 10. Treatment with opaganib alone modestly decreased the average tumor volume; however, the combination of IRIN + TMZ significantly reduced tumor growth relative to vehicle-treated mice. Interestingly, the addition of opaganib to IRIN + TMZ treatment further suppressed tumor growth compared to both the vehicle and the IRIN + TMZ groups, reaching high significance (*p* < 0.001) during the progression of the study. The growth of the Neuro-2a tumors was heterogeneous among individual mice in the treatment groups (the table in Figure 4). For the vehicle-treated group, 50% of the tumors grew very rapidly, reaching > 3000 mm^3^ by Day 10; whereas 30% remained below 1000 mm^3^ and 20% ranged from 1000–3000 mm^3^. The cause of this heterogeneity is not clear; however, opaganib alone and IRIN + TMZ shifted the pattern of growth toward slower progression, and the opaganib + IRIN + TMZ combination markedly suppressed the growth of all tumors. The median survival of the vehicle control group was 10 days; however, the median survivals for the IRIN + TMZ and opaganib + IRIN + TMZ treatment groups differed significantly from the vehicle control group (*p* = 0.016 and *p* = 0.012, respectively). In all, the three-drug combination of opaganib + IRIN + TMZ again was more efficacious than IRIN + TMZ treatment without increased toxicity. 

#### 3.2.3. Combination with Checkpoint Antibodies

We have previously demonstrated that opaganib promotes immunogenic cell death (ICD) in tumor cells, including Neuro-2a cells [45], which can enhance therapeutic responses to checkpoint antibodies. Therefore, we conducted studies on the antitumor activity of opaganib when combined with antibodies against either murine PD-1, PD-L1, or CLTA-4 toward Neuro-2a tumors growing in syngeneic A/J mice. In these experiments, opaganib was administered by oral gavage (50 mg/kg/day, 5 days/week), and anti-mouse PD-1, PD-L1, or CLTA-4 antibodies were injected intraperitoneally (200 μg) twice weekly for 3 weeks. In Experiment 1, treatment was initiated when tumors were small (~100 mm^3^); however, treatment in Experiment 2 was delayed until tumors were larger (~450 mm^3^) to more closely mimic clinical therapy. In both experiments, there was no significant toxicity from opaganib alone or in combination with the checkpoint antibodies. Single agent treatment with opaganib or the checkpoint antibodies generally reduced the average tumor size, but differences from controls were not statistically significant because of the heterogeneity in growth rates of the Neuro-2a tumors. Individual mice were euthanized when tumors reached >3000 mm^3^, and the median survival times for mice treated with vehicle, opaganib alone, checkpoint antibody alone, or opaganib plus checkpoint antibody are shown in Table 3. In both experiments, the survival advantage provided by the opaganib + anti-CTLA-4 antibodies was substantially greater than that of either drug alone, indicating that opaganib and anti-CTLA-4 antibodies have additive antitumor activity. In contrast, the combination of opaganib with anti-PD-1 or anti-PD-L1 antibodies did not increase antitumor activity over that seen with opaganib alone.

## 4. Discussion

Opaganib (previously called ABC294640) is the first clinical-stage drug targeting SK2 and DES1 for the treatment of cancer and pathologic inflammation. Opaganib depletes S1P and elevates ceramide in tumor cells, suppresses signaling through pERK and pAKT, and promotes autophagy and/or apoptosis in tumor cells [29,37,38,47]. We and others have shown that opaganib has broad antitumor activity in mouse models [37,39,48], which is associated with accumulation of opaganib in the tumors, depletion of tumor S1P levels, and induction of apoptosis [37]. Phase 1 clinical testing of opaganib given to patients with advanced solid tumors demonstrates that the drug is well-tolerated when administered orally on a twice-daily basis, continuously in 28-day cycles [49]. In this first-in-human trial, 64% of patients who completed two cycles of opaganib treatment had stable disease or better, suggesting that it has antitumor activity in most patients. As in animal models, opaganib decreased the patients’ plasma S1P over the first 12 hr, with a return to baseline at 24 h after a single dose. Plasma concentrations of opaganib peaked at 1–2 h, and declined with a mean half-time of 5–15 h. Doses of opaganib that have therapeutic efficacy in mouse xenograft models provide a C_max_ of ~3.5 μg/mL [37], a drug level that was achieved by 33%, 75%, and 100% of patients treated with 250, 500, and 750 mg of opaganib, respectively. In a second clinical trial of opaganib, 58% of patients with refractory multiple myeloma achieved stable disease or better, and patients had decreased plasma levels of TNFα, EGF, and VEGF [50]. Opaganib has also been assessed in hospitalized patients with severe COVID-19 and is completing Phase 2 clinical testing in patients with cholangiocarcinoma (ClinicalTrials.gov Identifier: NCT03377179) or prostate cancer (ClinicalTrials.gov Identifier: NCT04207255). Overall, opaganib has been given to almost 500 patients with an excellent safety profile and preliminary indications of anticancer and anti-COVID activity.

The potential for targeting sphingolipid metabolism as an NB treatment option has been considered by others [51]. Early studies demonstrated that ceramide inhibits NB cell growth and differentiation [52] and promotes NB apoptosis [53,54]. DES1 was shown to promote cell cycle progression in NB cells [55,56], and inhibition of this enzyme may be the basis for the cytotoxicity of fenretinide [55]. Fenretinide inhibits NB cell proliferation by inducing apoptosis [57,58,59] or lethal autophagy [60], and causes increases in dihydroceramides due to direct inhibition of DES1 [61], which is similar to opaganib. Activation of AKT suppresses ceramide-induced apoptosis in NB cells [62]. Importantly, NB cell lines and tissues express high levels of SK2 relative to SK1, and the resultant S1P promotes VEGF secretion from the cells [63]. Interesting data from Li et al. [64] demonstrate that FTY720 (known to be phosphorylated by SK2 [65]) downregulates SK2 expression and has antiproliferative and antitumor activity in NB cells. The involvement of sphingolipids in drug resistance in NB was also considered [66,67]. In addition to their direct effects on tumor cells, SKs regulate pathologic inflammation from cytokines such as TNFα and IL-6 [68,69,70]. In particular, production of S1P in response to inflammatory cytokines is dependent on SK activity [42,68,71,72,73,74,75,76,77,78,79,80,81,82,83,84], typically through NFκB [83]. NB tumors exist in a proinflammatory environment [85,86,87], and secrete high levels of chemokines and PGE_2_ that can promote tumor growth and progression [88]. IL-6 and VEGF promote NB growth and aggression [89,90], and opaganib is known to suppress the generation of these cytokines through inhibition of NFκB [91,92,93]. Therefore, altering sphingolipid metabolism, particularly targeting SK2 and DES1, appears to be a viable new approach to NB therapy.

Using a panel of mouse and human cell lines, we herein confirm that NB cells can be effectively killed by opaganib in vitro at concentrations that are clinically achievable. Importantly, cells possessing amplified *MYCN* are not less sensitive to opaganib-induced cytotoxicity than are single-copy *MYCN* cells. This contrasts with previous demonstrations that N-Myc and c-Myc decrease the sensitivity of tumor cells to many drugs [9,94,95,96]. Sphingolipid profiling confirmed inhibition of SK2 and DES1 in NB cells by opaganib and also indicated that production of hexosylceramides is also suppressed by the drug. Unfortunately, the LC-MS methods used in these analyses do not resolve glucosylceramide and galactosylceramide, so determining which specific hexosylceramide transferase is inhibited by opaganib is not yet possible. Glucosylceramides and galactosylceramides, although nearly identical structurally, have different tissue and cellular distributions, biological functions, and metabolisms than more complex sphingolipids (reviewed in [97,98]). Of note, both hexosyltransferases have been associated with increased tumor aggressiveness and metastasis. Sphingolipid profiling also indicated that opaganib reduces the levels of deoxyceramides, which are synthesized from alanine rather than serine and which have been linked to lipotoxicity and the progression to type 2 diabetes [99]. Because opaganib acts as a sphingosine mimetic, it is not surprising that it interacts with multiple enzymes in the sphingolipid pathway, specifically SK2, DES, glucosylceramide synthase, and/or galactosylceramide synthase.

Analyses of signaling protein expression confirmed that opaganib suppresses levels of Mcl-1, pERK, and c-Myc as in other cell types. This is the first report that opaganib also inhibits N-Myc in NB cells. The Myc proto-oncogene family (*MYC*, *MYCN*, and *MYCL*) encodes three closely related transcription regulatory proteins that have been widely studied as critical mediators of a variety of cell functions, including dysregulated cell proliferation, metabolism, and survival in cancer (reviewed in [100]). The importance of *MYCN* in the progression and pathology of NB has been well established [101]; however, high expression of c-Myc has also been shown to be associated with poor clinical outcomes in NB [102]. Interestingly, ablation of c-Myc by RNA interference inhibited the proliferation of *MYCN* single-copy cells, but not when N-Myc was overexpressed, indicating at least some functional compensation of c-Myc function in NB proliferation by N-Myc [103]. This is consistent with the high degree of sequence and structural similarities between c-Myc and N-Myc, which allow both proteins to interact with several partners, including MAX, which is required for DNA–protein interaction [100]. However, ablation of N-Myc in *MYCN*-amplified cells suppressed proliferation and induced apoptosis, indicating that c-Myc is not sufficient for the survival of *MYCN*-driven NB cells [104]. As one mechanism for promoting tumor cell survival, N-Myc promotes the expression of telomerase [105], and we have previously shown that opaganib downregulates telomerase expression [106]. Overall, it seems optimal to attenuate the expression of both c-Myc and N-Myc in NB patients with tumors of mixed or unknown status of *MYCN* amplification. Previous data showing that opaganib downregulates c-Myc levels [48] and the current data demonstrating N-Myc downregulation support the postulate that this drug may be particularly effective for NB therapy. Beyond tumor cell proliferation and survival, N-Myc has been implicated in metastasis [101,107], which is present in approximately half of newly diagnosed NB patients [108]. It would therefore be useful to also examine the anti-metastatic potential of opaganib in a NB tumor model that allows tumor migration, such as that described by Seong et al., in which the dual SK1/SK2 inhibitor SKI-II suppressed NB cell migration in vivo [109].

In vivo models confirmed that single-agent opaganib suppresses the growth of NB tumors from single-copy *MYCN* cells (Neuro-2a) and amplified *MYCN* cells (SK-N-(BE)2). We focused on studies with syngeneic tumor cells so that immunocompetent mice could be used as hosts. Cancer chemotherapy typically involves the administration of multiple drugs that are selected for non-overlapping mechanisms of action, resistance, and toxicity. Therefore, we evaluated the antitumor activity of opaganib in combination with irinotecan (IRIN) plus temozolomide (TMZ) because IRIN + TMZ is the backbone for treatment of recurrent or refractory NB alone or in combination with additional potential therapies [110,111]. C57BL/6 mice bearing LLC tumors were selected for the initial exploratory study because of their established tumor growth rates and responsiveness to opaganib [45]. This study demonstrated a significantly increased efficacy of opaganib + IRIN + TMZ relative to vehicle- and IRIN +TMZ-treated mice. This was then confirmed in studies of Neuro-2a cells growing in syngeneic A/J mice. In this model, IRIN + TMZ has substantial antitumor activity, and this was significantly further improved when opaganib was added to the combination. Importantly, both models demonstrated that there is no significant toxicity for the combination of opaganib + IRIN +TMZ. Taken together, the tumor models support the clinical evaluation of opaganib in combination with IRIN + TMZ in NB patients. 

Immunotherapy using antibodies that target the checkpoint proteins CTLA-4, PD-1, and PD-L1 is improving the treatment of some cancers; however, combination therapies that will provide broader and more sustained clinical responses are desired. Finally, we conducted studies of the antitumor efficacy of opaganib in combination with antibodies against the checkpoint regulators CTLA-4, PD-1, and PD-L1. Despite limited success in NB therapy, targeting checkpoint proteins continues to be explored in clinical trials of this disease [27]. We have previously demonstrated that opaganib promotes immunogenic cell death (ICD) in tumor cells, including Neuro-2a cells [45], which can enhance therapeutic responses to checkpoint antibodies. Additionally, both c-Myc [112] and N-Myc [113] have been shown to modulate anti-tumor immune suppression, and so a combination of Myc inhibitors with cancer immunotherapy may improve NB patient survival. Therefore, we conducted studies on the antitumor activity of opaganib when combined with antibodies against either murine PD-1, PD-L1, or CLTA-4 toward Neuro-2a tumors growing in syngeneic A/J mice. The survival advantage provided by the opaganib + anti-CTLA-4 antibodies was substantially greater than either drug alone, indicating that opaganib and anti-CTLA-4 antibodies have additive antitumor activity. In contrast, the combination of opaganib with anti-PD-1 or anti-PD-L1 antibodies did not increase antitumor activity over that seen with opaganib alone. Therefore, we believe there is potential benefit in the combination of opaganib with anti-CTLA-4 antibodies for the treatment of NB patients.

We recognize that additional work will be useful in defining several aspects of the data presented in this initial report. For example, additional in vivo models using NB cells with either single-copy or amplified *MYCN* would be useful for confirming the activity of opaganib against both types of tumors. There are, however, a few murine NB cell lines that would be needed for studies in immunocompetent mice. Additionally, the effects of opaganib on tumor infiltration by immune cells and the expression of checkpoint proteins in both the tumors and immune cells would be helpful in further determining the potential for a combination of opaganib and checkpoint antibodies in NB therapy.

In summary, opaganib treatment effectively inhibits SK2, DES1, and hexosylceramide synthase and depletes N-Myc as well as c-Myc proteins from NB cells. To our knowledge, this is the only drug shown to have this range of activity, and this results in anticancer activity manifested in vivo as suppression of NB tumor growth by opaganib alone or in combination with the standard drugs irinotecan and temozolomide. Additionally, opaganib promotes immunogenic cell death in NB cells, which results in enhanced antitumor activity when it is combined with anti-CTLA-4 antibody therapy. Overall, the data support our hypothesis that NB may be a particularly good disease for clinical trials of opaganib, particularly in combination with other cytotoxic or immunomodulatory drugs.

## Figures and Tables

**Table 1:** Cytotoxicity of opaganib toward NB cells. Cells were incubated with varying concentrations of opaganib for 72 h, and the surviving fraction was determined. Values indicate the IC_50_ mean ± SEM (*n* = 2–8, except for IMR32, where *n* = 1).

Cell Line	*MYCN* Status	Replicates	IC_50_ for Opaganib (μM)
Neuro-2a	Single	8	7.5 ± 1.5
SK-N-SH	Single	2	35.0 ± 2.0
SK-N-AS	Single	2	19.8 ± 5.3
SK-N-MC	Single	2	16.0 ± 6.0
IMR32	Amplified	1	7.6
SK-H-(BE)2	Amplified	4	18.5 ± 3.2

**Table 2:** Alteration of sphingolipid profiles in Neuro-2a cells treated with opaganib. Cells were incubated with the indicated concentrations of opaganib for 24 h, and sphingolipid profiles were quantified by LC-MS. The top row indicates the absolute mass of the indicated lipid(s) and the later rows are expressed relative to the mass in the vehicle-treated control cells. Values represent the mean of triplicate samples.

Opaganib (μM)	Sphingosine	S1P	Total Ceramides	Total Dihydroceramides	Total Deoxyceramides	Total Hexosylceramides
0	186 pmol/mg	5.1 pmol/mg	1211 pmol/mg	41 pmol/mg	59 pmol/mg	1492 pmol/mg
0	100	100	100	100	100	100
1	81	80	103	116	87	89
3	94	81	112	148	97	90
10	90	87	98	165	92	93
50	22	7	87	333	47	12

**Table 3:** Effect of opaganib in combination with checkpoint antibodies on the survival of mice bearing Neuro2a tumors. Individual mice were sacrificed when the tumor volume exceeded 3000 mm^3^. The median survival in days is shown.

	Experiment 1 Starting Tumor Volume = 106 ± 30 mm^3^		Experiment 2 Starting Tumor Volume = 460 ± 33 mm^3^	
Treatment	Median Survival (Days)	Change from Control (Days)	Median Survival (Days)	Change from Control (Days)
Vehicle	24	-	9	-
Opaganib alone	34	10	18.5	9.5
Anti-CTLA-4	34	10	21.5	12.5
Opaganib + anti-CTLA-4	43.5	19.5	30	21
Anti-PD-1	31	7	17	8
Opaganib + Anti-PD-1	35	11	31	22
Anti-PD-L1	31	7	17	8
Opaganib + anti-PD-L1	34	10	14.5	5.5

## Data Availability

All data is presented in the published paper.

## Supplementary Materials

The following supporting information can be downloaded at: https://www.mdpi.com/article/10.3390/cancers16091779/s1, Figure S1: Uncropped Western Blot figures.
