# Supplementary figures and images for: Opaganib Downregulates N-Myc Expression and Suppresses In Vitro and In Vivo Growth of Neuroblastoma Cells
Source: Cancers (Basel). 2024 May 5;16(9):1779. doi: 10.3390/cancers16091779 (PMC11082966; doi:10.3390/cancers16091779)

Figure S1 c-Myc

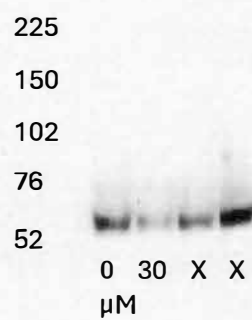

Figure S1 ERK

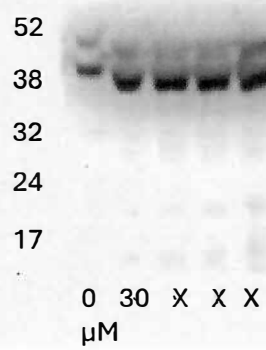

Figure S1 GAPDH

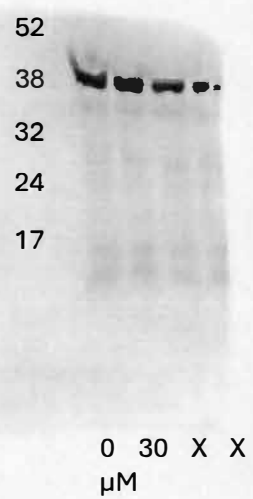

Figure S1 Mcl-1

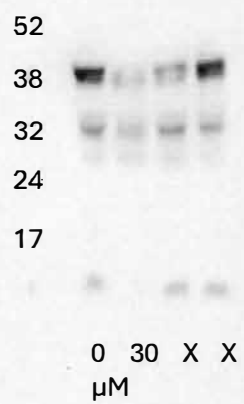

Figure S1 N-Myc

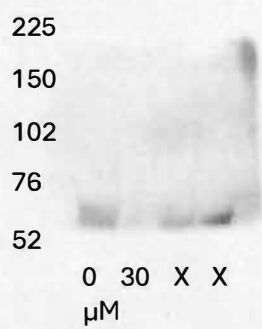

Figure S1 pERK

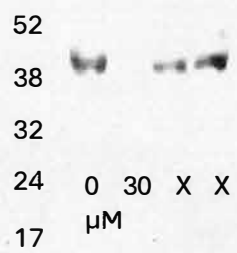

Supplement: Supplementary file 1 [file cancers-16-01779-s001.zip › cancers-2940550-supplementary.pdf]
